# Profiles of HIV Care Disruptions Among Adult Patients Lost to Follow-up in Zambia: A Latent Class Analysis

**DOI:** 10.1097/QAI.0000000000002530

**Published:** 2021-10-16

**Authors:** Aaloke Mody, Kombatende Sikombe, Laura K. Beres, Sandra Simbeza, Njekwa Mukamba, Ingrid Eshun-Wilson, Sheree Schwartz, Jake Pry, Nancy Padian, Charles B. Holmes, Carolyn Bolton-Moore, Izukanji Sikazwe, Elvin H. Geng

**Affiliations:** a Division of Infectious Diseases, Washington University School of Medicine, St. Louis, MO;; b Centre for Infectious Diseases Research in Zambia, Lusaka, Zambia;; c Department of Public Health Environments and Society, Faculty of Public Health and Policy, London School of Hygiene and Tropical Medicine, London, United Kingdom;; d Department of International Health, Johns Hopkins University Bloomberg School of Public Health, Baltimore, MD;; e Division of Epidemiology, University of California, Berkeley, Berkeley, CA;; f Department of Medicine, Georgetown University, Washington, DC; and; g Division of Infectious Diseases, University of Alabama, Birmingham, AL.

**Keywords:** latent class analysis, phenotypes, barriers to care, retention in care, loss to follow-up, transfer, disengagement

## Abstract

**Background:**

Patients report varied barriers to HIV care across multiple domains, but specific barrier patterns may be driven by underlying, but unobserved, behavioral profiles.

**Methods:**

We traced a probability sample of patients lost to follow-up (>90 days late) as of July 31, 2015 from 64 clinics in Zambia. Among those found alive, we ascertained patient-reported reasons for care disruptions. We performed latent class analysis to identify patient subgroups with similar patterns of reasons reported and assessed the association between class membership and care status (ie, disengaged versus silently transferred to a new site).

**Results:**

Among 547 patients, we identified 5 profiles of care disruptions: (1) **“Livelihood and Mobility”** (30.6% of the population) reported work/school obligations and mobility/travel as reasons for care disruptions; (2) **“Clinic Accessibility”** (28.9%) reported challenges with attending clinic; (3) **“Mobility and Family”** (21.9%) reported family obligations, mobility/travel, and transport-related reasons; (4) **“Doubting Need for HIV care”** (10.2%) reported uncertainty around HIV status or need for clinical care, and (5) **“Multidimensional Barriers to Care”** (8.3%) reported numerous (mean 5.6) reasons across multiple domains. Patient profiles were significantly associated with care status. The “**Doubting Need for HIV Care**” class were mostly disengaged (97.9%), followed by the **“Multidimensional Barriers to Care”** (62.8%), **“Clinic Accessibility”** (62.4%), **“Livelihood and Mobility”** (43.6%), and **“Mobility and Family”** (23.5%) classes.

**Conclusion:**

There are distinct HIV care disruption profiles that are strongly associated with patients' current engagement status. Interventions targeting these unique profiles may enable more effective and tailored strategies for improving HIV treatment outcomes.

## Abstract 2

Supplemental Digital Content is Available in the Text.

## INTRODUCTION

Despite tremendous scale-up of HIV treatment in sub-Saharan Africa over the past decade, HIV treatment outcomes remain suboptimal, in large part because of poor retention in care.^1^ Effective strategies to improve engagement are urgently needed as upward of 25% of patients will disengage from care.^2,3^ There is also increasing recognition that these strategies—as opposed to being uniform and directed toward the entire patient population—will also need to be more targeted to account for the significant diversity in the patient population.^4–6^ It is thus imperative that we develop a more comprehensive understanding of the heterogeneity in the HIV patient population to develop such targeted strategies to optimize the impact of the global HIV treatment response.

Although patients report a diversity of individual barriers, individual patients may also experience specific patterns of barriers—defined by individual-, social-, and systems-level characteristics—that ultimately drive their behavior. Existing approaches that use a priori (even if theory-based) categories to describe barriers do not make use of the data itself to empirically explore these groupings. Latent class analysis (LCA) is a data-driven approach to identify latent (or unobserved) subgroups hidden within the data, thereby revealing coherent groupings that potentially point to mechanisms of behavior.^7^ In leveraging multi-dimensional data to identify subgroups with unique data patterns, LCA can capture potentially complex associations and interaction between different variables. Rather than targeting interventions based on only a single sociodemographic characteristic (eg, age or sex) or risk factors (eg, stigma or food insecurity), LCA can identify patient profiles that may better capture the heterogeneity in behavioral patterns and risk profiles. Ultimately, focusing on more holistic profiles rather than single characteristics may be a more effective strategy for developing and targeting interventions for a diverse patient population.

We sought to identify unique patient profiles of HIV care disruptions in Zambia using latent class methods. We used LCA and data on patient-reported reasons for loss to follow-up among a population-representative sample of patients in Zambia to identify profiles of HIV care disruptions and examined their associations with engagement in care status. This in-depth characterization of patient profiles ultimately permits a better understanding of the drivers of patient behaviors and clearly lays forth actionable targets for improving retention in care.

## METHODS

### Patient Population and Setting

We analyzed a cohort of adults (18 years old or older) living with HIV on antiretroviral therapy (ART) who were lost to follow-up (LTFU, defined as being greater than 90 days late to a scheduled appointment or 180 days from any recorded clinic encounter if no scheduled appointment was found) from public health clinics in Zambia and who were then found alive after actively being traced in the field. Patients were identified as part of the *Better Information for Health in Zambia* study, in which we used previously validated methods to undertake multistage random sampling and tracing patients in the community to obtain population-representative estimates of retention and mortality.^8–10^ We first identified all patients who had made at least one clinic visit after August 1, 2013 but who were LTFU as of July 31, 2015 from 64 health facilities in 4 of Zambia's 10 provinces. In the first sampling stage, we selected a stratified random sample of 32 of the 64 health facilities to conduct tracing activities. At these 32 selected facilities, we then identified a random sample of the patients who were LTFU. These patients were then traced between September 2015 and July 2016. Study clinics are operated by the Zambian Ministry of Health and receive technical support from the Centre for Infectious Disease Research in Zambia, a Zambian non-governmental organization.

### Measurements

We asked patients who were found alive during phone or in-person tracing and self-reported an HIV care disruption (ie, either currently out of care or transferred to another facility after disengaging from their original facility) an open-ended question about why their care was disrupted (“Why did you stop going to any clinic for your HIV care?” or “Why are you going to your new clinic instead of your previous one?” depending on current care status). Patients responded to the open-ended prompt and the reasons they reported were then recorded by trained interviewers using prespecified categories [which were identified based on literature review, input from key informants, and prior experience using this method to ascertain patient-reported barriers to care (see Table 1, Supplemental Digital Content, http://links.lww.com/QAI/B555)].^11^ We obtained sociodemographic (eg, age, sex, clinic site), clinical (date of ART initiation, CD4 counts), and visit history (dates, scheduled appointment) measurements from the national electronic medical record system used in routine HIV care in Zambia. To populate the electronic medical record, providers use standardized paper clinical forms during patient encounters that are then entered into the electronic database by data clerks retrospectively.

### Analyses

#### Latent Class Analysis

We performed LCA to identify distinctive patient profiles of care disruptions using data on patient-reported reasons for LTFU. This method is based on the premise that the population is made of unique, but unobserved, subpopulations that have distinct patterns in their data, and it uses the observed data to identify what these unique patterns are, which patients may belong to a particular subgroup, and how these subgroups are distributed in the population.^7^ We used LCA to identify subgroups of LTFU patients that had similar patterns in both the types of reasons patients reported for their care disruption and the total number of reasons they reported. Because there were initially 65 possible reasons for care disruptions, we undertook an initial round of data reduction based on theory, literature review, and contextual experience to combine similar reasons into 15 categories that could then be analyzed using LCA (see Tables 1 and 2, Supplemental Digital Content, http://links.lww.com/QAI/B555). We then performed LCA to estimate both the pattern of reasons for care disruptions for each class (ie, the probability of reporting particular reasons and the mean number of reasons reported) and the percent of patients expected to belong to each class. Since the number of groups is not known a priori, we systematically tested models with differing number of groups and then selected a final model that was optimized for fit and parsimony—using the Akaike Information Criterion and Bayesian Information Criterion in text—and interpretability—using contextual knowledge.^7,12^ Based on this final model, we then estimated each individual's probability of belonging to a specific latent class given their reported reasons (ie, estimated posterior probabilities based on Bayes Theorem) and assigned them to the latent class to which they were most likely to belong (ie, the maximal probability rule).^12^

We assessed adequacy and fit of the final model and group assignment using several established metrics: (1) comparing the proportion assigned to each latent class using maximal probability rule versus the estimated distribution from the initial model, (2) estimating the average posterior probability for individuals assigned to each class using the maximal probability rule, (3) calculating the odds ratio of being assigned to the correct latent class based on posterior probabilities, and (4) calculating the entropy statistic, an indicator of separation between latent classes.^7,12^

#### Baseline Factors Associated with Latent Class

We described baseline patient sociodemographic, clinical, and facility-level characteristics for members of each latent class (using latent class assignments based on maximum posterior probabilities). In addition, we used multinomial logistic regression to assess for patient characteristics associated with class membership and then estimated the predictive margins of the expected distribution of latent classes stratified across each characteristic.

#### Association Between Latent Class and Care Status

Finally, we sought to assess the association between latent class membership and whether patients remained out of care or had silently transferred to a new facility. We performed unadjusted and adjusted Poisson regression with robust variances and estimated the marginal prevalence of being out of care. Adjusted models included latent class membership and sociodemographic, clinical, and facility-level characteristics based on directed acyclic graphs. We also assessed for an interaction between latent class and sex.

All analyses incorporated sampling weights based on the inverse of the probability of being selected for tracing to yield population-representative estimates.^8–10^ We used multiple imputation (n = 20) to address missingness in predictor variables (ie, enrollment CD4 count, enrollment WHO Stage, marital status, and education status).^13^ As an individual's class membership is not observed and only predicted based on individuals' own observed data, we also conducted sensitivity analyses that account for potential misclassification arising from the uncertainty in latent class assignments (see Appendix 1, Supplemental Digital Content, http://links.lww.com/QAI/B555).^12,14^ All analyses were conducted using Stata (Version 16.1, College Station, TX). The study was approved by the University of Zambia Biomedical Research Ethics Committee (UNZABREC) and institutional review boards at the University of California, San Francisco and the University of Alabama, Birmingham School of Medicine. All participants provided informed consent before being interviewed.

## RESULTS

### Patient Characteristics

**FIGURE 1.**
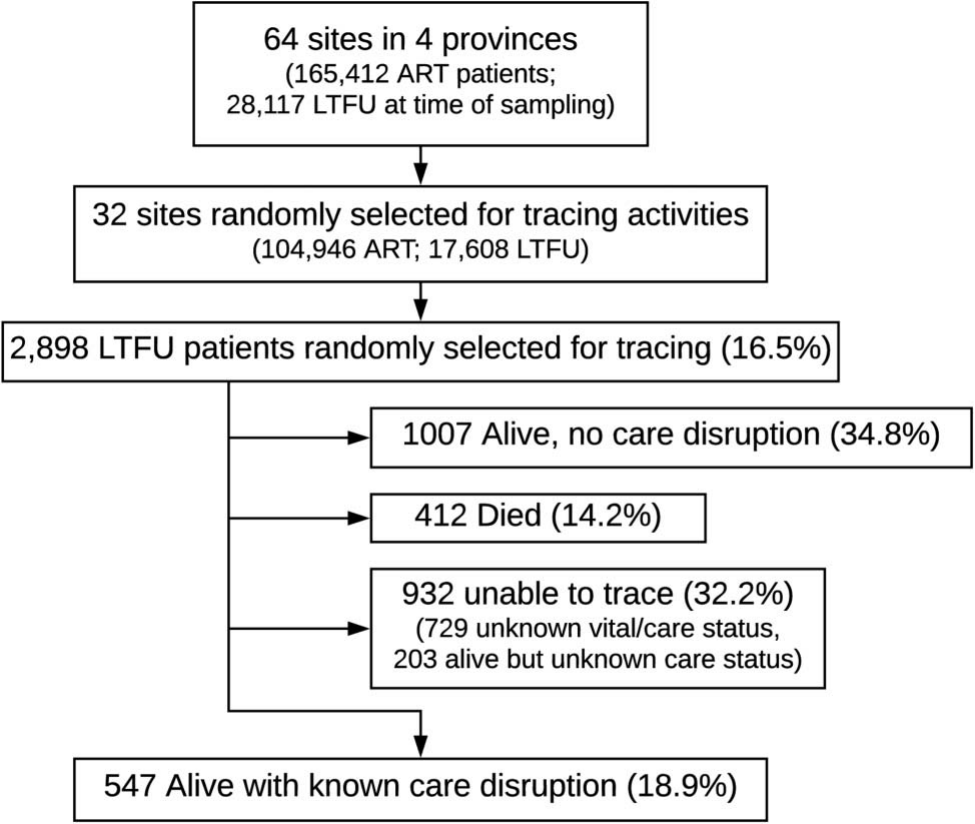
Patient flowchart. As of July 1, 2015, 28,117 patients who had ever initiated ART were considered LTFU across 64 sites and 2898 were randomly selected for active tracing from 32 sites. Among patients selected for tracing, 1007 (34.8%) were found alive without a care disruption, 412 (14.2%) had died, and we were unable to trace 932 (32.2%). We ascertained patient-reported reasons for care disruptions among the 547 (18.9%) patients who we found alive with a confirmed care disruption.

After multistage sampling and active tracing of selected patients in the community, we interviewed 547 participants who were found alive and in-person regarding reasons for their care disruption (Fig. 1). 326 (59.6%) patients were female the median age at the time of LTFU was 35 years (IQR 30–41), and the median time from ART initiation to LTFU was 1.3 years (IQR 0.3–3.5) (Table 1). The patient-reported reasons for LTFU are presented in Table 2, Supplemental Digital Content, http://links.lww.com/QAI/B555. At the time of tracing, 255 (46.6%) reported being out care, whereas 292 (54.4%) reported having transferred to a new facility since their last visit at their original facility (Table 1).

**TABLE 1.** Baseline Patient Characteristics

Baseline Patient Characteristics, n = 547	
Female sex, n (%)	326 (59.6%)
Median age at LTFU, yrs (IQR)	35 (30–41)
Median enrollment CD4 count*, cells/μL (IQR)	239 (130–366)
Enrollment WHO stage, n (%)	
I	234 (42.8%)
II	93 (17.0%)
III	137 (25.0%)
IV	31 (5.7%)
Unknown	52 (9.5%)
Median time from enrollment to ART initiation, d (IQR)	27 (14–85)
Median time from ART initiation to LTFU, yrs (IQR)	1.3 (0.3–3.5)
Prior episodes of LTFU, n (%)	
0	290 (53.0%)
1	165 (30.2)
2	63 (11.5)
>3	29 (5.3%)
Marital status, n (%)	
Single	105 (19.2%)
Married	315 (57.6%)
Divorced	74 (13.5%)
Widowed	49 (9.0%)
Unknown	4 (0.7%)
Education, n (%)	
None	31 (5.7%)
Lower–Mid basic	199 (36.4%)
Upper basic/Secondary	243 (44.4%)
College/University	67 (12.2%)
Unknown	7 (1.3%)
Disclosed HIV status, n (%)	
Yes	472 (86.3%)
No	10 (1.8%)
Unknown	65 (11.9%)
Facility type	
Urban	308 (56.3%)
Rural	100 (18.3%)
Hospital	139 (25.4%)
Province, n (%)	
Lusaka	235 (43.0%)
Eastern	94 (17.2%)
Southern	123 (22.5%)
Western	95 (17.4%)
Care status at time of interview, n (%)	
Disengaged	255 (46.6%)
Silently transferred	292 (53.4%)

* Missing for 103 patients.

IQR, interquartile range; LTFU, loss to follow-up.

### Description of Latent Classes

**FIGURE 2.**
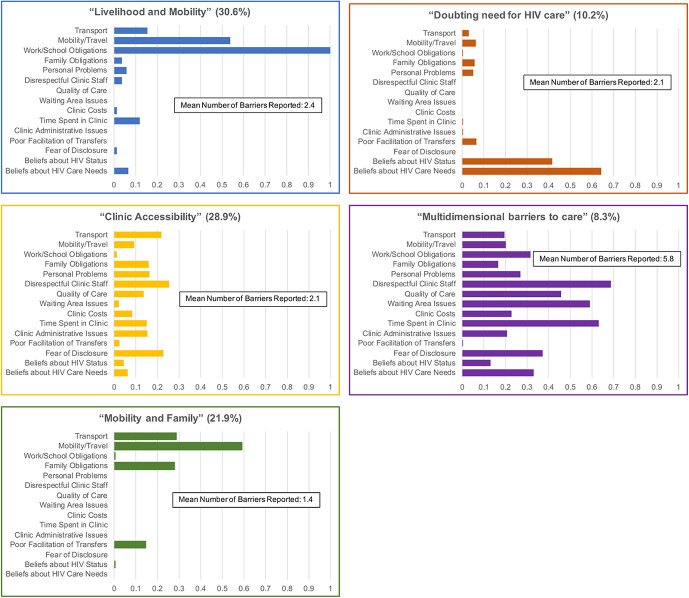
Profiles of care disruptions (n = 547). Patient profiles of care disruption are based on latent class models based on the patient-reported reasons for care disruptions and the number of reasons a patient reported. The estimated proportion of patients in each latent class are in parentheses at the top, and bars correspond to the probability of reporting a particular reason for care disruption within each class. Models used population-representative sampling weights after tracing a random sample of patients who were considered lost to follow-up as of July 31, 2015.

We selected the model with 5 latent classes (ie, profiles of care disruptions) based on model fit and interpretability (Fig. 2). In this model, the first class were patients who reported predominately work/school obligations and mobility/travel challenges (mean 2.4 reasons) as reasons for their care disruptions (“Livelihood and Mobility” group) and accounted for 30.6% [95% confidence interval (CI): 25.5% to –36.3%] of the population. The second class primarily reported various issues (mean 2.1) associated with attending clinic (eg, rude health care workers, quality of care, time spent in clinic) [“Clinical Accessibility” group, 28.9% (95% CI: 20.2% to 39.4%)]. The third class reported mobility/travel, family obligations, and transport issues (mean 1.4) as reasons for care disruptions [“Mobility and Family” group, 21.9% (95% CI: 16.3% to 28.8%) of the population]. The fourth class were patients who reported doubts regarding their HIV status or need to routinely come to clinic (mean 2.1) [“Doubting need for HIV Care” group, 10.2% (95% CI: 5.6% to –18.0%)]. Finally, the fifth class were patients who reported numerous (mean 5.6) issues across multiple domains (structural, clinic-level, and psychosocial) as reasons for their care disruption (“Multidimensional Barriers to Care” group, 8.3% [95% CI: 3.3%–19.4%] of the population) (Fig. 2, and Table 3, Supplemental Digital Content, http://links.lww.com/QAI/B555). Diagnostic metrics demonstrate that our final model had very good fit and separation between classes (Table 2).

**TABLE 2.** Metrics of Adequacy and Fit of Latent Class Model

Metrics of Adequacy and Fit of Latent Class Model					
	Group Average Posterior Probability	Odds Ratio for Correct Classification	Estimated Group Distribution Based on Using Maximal Probability Rule	Estimated Group Distribution Based on Initial Model	Entropy
Livelihood and mobility	0.983	128	0.311	0.306	0.858
Clinic accessibility	0.940	48	0.247	0.289	
Mobility and family	0.811	12	0.270	0.219	
Doubting need for HIV care	0.899	80	0.100	0.102	
Multidimensional barriers to care	0.951	252	0.072	0.083	

Good model fit indicated by 1) average posterior probability greater than 0.7 for each group, 2) odds ratio of correct classification greater than 5 for each group; 3) close correspondence between the estimated group distribution based on using posterior probabilities and the maximal probability rule compared with the estimated group distribution from the initial model; and 4) an entropy greater than 0.8.

### Baseline Factors Associated With Latent Class Membership

There were several notable trends when examining baseline characteristics associated with latent class membership, although few were strongly predictive of class membership (Tables 3 and 4). The “Livelihood and Mobility” class trended toward being male, having more prior episodes of LTFU, and being from Lusaka province (which includes the capital city and surrounding areas). Patients with “Clinic Accessibility” problems were more likely to be women, from urban areas, and have more advanced disease at enrollment. The “Mobility and Family” class trended toward having no prior LTFU and were less likely to be from Lusaka. Those “Doubting the Need for HIV Care” trended toward having lower WHO stage, being either earlier in their care (ie, LTFU occurred within 6 months of ART initiation) or with over 3 prior episodes of LTFU, being widowed, and having lower education. Finally, patients with “Multidimensional Barriers to Care” trended toward being male, single, more educated, from rural areas, and having had longer periods in care with multiple episodes of prior LTFU.

**TABLE 3.** Baseline Patient Characteristics by Latent Class

Baseline Patient Characteristics by Latent Class, n = 547					
	Livelihood and Mobility	Clinic Accessibility	Mobility and Family	Doubting Need for HIV Care	Multidimensional Barriers to Care
Female sex, percent	47.4%	65.6%	64.4%	58.2%	52.0%
Median age at LTFU, yrs (IQR)	37 (31–41)	36 (30–42)	35 (28–40)	36 n(31–42)	36 (32–39)
Median enrollment CD4 count, cells/μL (IQR)	213 (125–340)	214 (130–317)	236 (106–397)	217 (118–407)	167 (113–328)
Enrollment WHO stage, percent					
I	43.0%	46.7%	55.7%	63.7%	43.1%
II	17.9%	17.5%	12.3%	19.3%	18.8%
III	30.8%	32.1%	27.3%	15.6%	34.4%
IV	8.3%	3.7%	4.7%	1.4%	3.7%
Median time from ART initiation to LTFU, yrs (IQR)	2.0 (0.6–4.2)	1.4 (0.5–2.9)	0.9 (0.1–2.7)	0.2 (0.0–1.6)	2.7 (0.3–4.0)
Prior episodes of LTFU, percent					
0	34.8%	52.7%	58.2%	68.8%	51.5%
1	37.3%	38.6%	28.4%	19.5%	27.7%
2	21.9%	5.4%	9.2%	3.5%	9.5%
>3	6.0%	3.4%	4.2%	8.1%	11.3%
Marital status, percent					
Single	19.4%	13.8%	21.0%	19.8%	29.3%
Married	63.5%	55.3%	58.2%	53.1%	45.6%
Divorced	11.5%	17.2%	15.0%	9.7%	19.6%
Widowed	5.6%	13.8%	5.8%	17.3%	5.5%
Education, percent					
None	2.2%	3.4%	5.2%	8.5%	0.5%
Lower–Mid basic	31.6%	42.7%	27.8%	38.5%	31.0%
Upper basic/Secondary	41.2%	43.0%	53.3%	33.0%	44.8%
College/University	25.0%	10.8%	13.6%	20.0%	23.6%
Facility type, percent					
Urban	75.1%	75.2%	67.1%	79.6%	67.5%
Rural	3.9%	4.5%	6.2%	6.6%	13.8%
Hospital	21.1%	20.3%	26.7%	13.8%	18.7%
Province, percent					
Lusaka	74.6%	68.5%	56.6%	72.0%	71.4%
Eastern	5.4%	9.1%	14.5%	9.7%	4.5%
Southern	6.3%	12.2%	15.8%	14.2%	15.8%
Western	13.8%	10.2%	13.1%	4.2%	8.4%

All values calculated accounting for sampling weights included in the initial latent class model.

IQR, interquartile range; LTFU, loss to follow-up.

**TABLE 4.** Predictive Margins of Latent Class Distribution Across Baseline Patient Characteristics From Multinomial Logistic Regression

Predictive Margins of Latent Class Distribution Across Baseline Patient Characteristics from Multinomial Logistic Regression, n = 547					
	Livelihood and Mobility	Clinic Accessibility	Mobility and Family	Doubting Need for HIV Care	Multidimensional Barriers to Care
Overall	30.6% (25.5% to –36.3%)	28.9% (20.2% to –39.4%)	21.9% (16.3% to –28.8%)	10.2% (5.6% to –18.0%)	8.3% (3.3% to –19.4%)
Sex					
Female	27.1% (20.9% to –33.4%)	29.2% (22.8% to –35.5%)	28.3% (22.0% to –34.7%)	8.9% (5.4% to –12.4%)	6.5% (2.9% to –10.1%)
Male	36.2% (28.1% to –44.3%)	18.8% (12.5% to –25.2%)	24.7% (17.0% to –32.4%)	12.1% (6.5% to –17.8%)	8.1% (3.4% to –12.9%)
Age at LTFU					
<25 yrs old	23.3% (9.3% to –37.4%)	25.8% (11.6% to –40.0%)	35.5% (18.9% to –52.2%)	10.7% (0.7% to –20.7%)	4.7% (0.0% to –10.6%)
25–35 yrs old	34.2% (26.1% to –42.4%)	23.6% (16.9% to –30.3%)	24.7% (17.4% to –32.0%)	9.8% (4.8% to –14.7%)	7.7% (3.1% to –12.4%)
35–50 yrs old	30.3% (23.1% to –37.6%)	24.3% (17.5% to –31.0%)	25.4% (18.5% to –32.4%)	11.2% (6.2% to –16.2%)	8.8% (4.0% to –13.5%)
>50 yrs old	29.2% (9.1% to –49.3%)	29.9% (13.3% to –46.5%)	36.3% (16.6% to –55.9%)	4.7% (0.0% to –10.2%)	0.0% (0.0% to –0.0%)
Enrollment CD4 count					
<200 cells/μL	28.0% (21.3% to –34.6%)	27.0% (19.7% to –34.2%)	26.8% (19.8% to –33.9%)	10.6% (5.1% to –16.0%)	7.7% (3.3% to –12.0%)
200–350 cells/μL	33.5% (23.9% to –43.0%)	29.6% (20.7% to –38.5%)	23.0% (14.7% to –31.2%)	7.8% (3.2% to –12.4%)	6.2% (1.4% to –11.1%)
350–500 cells/μL	37.5% (24.0% to –51.0%)	13.3% (5.0% to –21.5%)	30.9% (17.4% to –44.4%)	11.0% (2.6% to –19.4%)	7.3% (0.7% to –14.0%)
>500 cells/μL	31.2% (16.1% to –46.4%)	16.8% (5.9% to –27.7%)	32.5% (17.5% to –47.6%)	12.1% (1.7% to –22.6%)	7.4% (0.0% to –15.2%)
WHO stage					
I	29.5% (22.5% to –36.6%)	21.3% (14.9% to –27.8%)	29.7% (22.2% to –37.2%)	12.1% (7.1% to –17.1%)	7.3% (2.7% to –11.9%)
II	37.5% (23.8% to –51.2%)	25.3% (14.3% to –36.3%)	18.2% (9.2% to –27.3%)	11.9% (3.3% to –20.6%)	7.1% (1.0% to –13.1%)
III	28.4% (19.5% to –37.3%)	30.8% (20.5% to –41.0%)	26.9% (17.0% to –36.9%)	5.9% (1.8% to –9.9%)	8.0% (2.9% to –13.2%)
IV	41.7% (17.9% to –65.4%)	23.4% (1.8% to –45.0%)	28.9% (7.6% to –50.2%)	2.8% (0.0% to –8.1%)	3.3% (0.0% to –7.5%)
Time to LTFU					
<6 mo	31.3% (19.7% to –42.9%)	17.7% (9.8% to –25.6%)	28.3% (19.1% to –37.6%)	16.9% (7.8% to –26.0%)	5.7% (1.3% to –10.2%)
6 months-2 yrs	30.1% (20.6% to –39.7%)	33.6% (23.9% to –43.3%)	22.4% (14.7% to –30.1%)	10.1% (4.5% to –15.8%)	3.7% (0.0% to –8.5%)
2–5 yrs	29.5% (19.4% to –39.6%)	26.6% (17.0% to –36.3%)	27.6% (17.1% to –38.1%)	1.9% (0.2% to –3.6%)	14.4% (4.5% to –24.2%)
>5 yrs	35.4% (21.6% to –49.2%)	17.6% (7.4% to –27.7%)	32.8% (17.2% to –48.5%)	7.6% (0.8% to –14.4%)	6.7% (0.0% to –14.3%)
Prior episodes of LTFU					
0	22.1% (14.8% to –29.5%)	29.2% (21.1% to –37.3%)	30.7% (22.6% to –38.8%)	9.3% (5.7% to –12.9%)	8.7% (3.0% to –14.4%)
1	35.9% (25.1% to –46.7%)	24.3% (16.8% to –31.8%)	24.1% (15.7% to –32.6%)	10.0% (2.9% to –17.1%)	5.7% (1.6% to –9.8%)
2	54.2% (37.5% to –70.9%)	12.2% (2.3% to –22.1%)	21.5% (9.0% to –34.0%)	8.3% (0.0% to –18.3%)	3.8% (0.1% to –7.6%)
>3	25.9% (7.1% to –44.8%)	12.3% (0.0% to –24.8%)	15.5% (0.0% to –32.0%)	33.2% (6.9% to –59.5%)	13.1% (0.0% to –28.0%)
Marital status					
Single	35.1% (23.9% to –46.3%)	17.5% (8.6% to –26.4%)	25.7% (15.5% to –35.9%)	10.8% (2.4% to –19.1%)	10.9% (1.3% to –20.5%)
Married	32.8% (26.2% to –39.5%)	24.9% (18.7% to –31.1%)	27.9% (21.9% to –33.9%)	9.0% (5.2% to –12.8%)	5.4% (2.6% to –8.1%)
Divorced	23.9% (12.3% to –35.5%)	25.5% (14.6% to –36.4%)	32.4% (18.9% to –45.9%)	7.8% (0.0% to –15.9%)	10.3% (2.0% to –18.6%)
Widowed	22.6% (7.8% to –37.5%)	35.7% (18.9% to –52.5%)	16.9% (5.1% to –28.7%)	17.8% (5.3% to –30.2%)	7.0% (0.0% to –17.3%)
Education					
None	21.2% (−5.0% to 47.3%)	21.9% (3.3% to –40.4%)	31.9% (9.6% to –54.3%)	23.9% (0.0% to –47.9%)	1.1% (0.0% to –6.7%)
Lower–Mid basic	29.2% (20.6% to –37.8%)	31.0% (22.3% to –39.7%)	21.6% (14.6% to –28.5%)	11.7% (5.5% to –17.9%)	6.5% (2.7% to –10.4%)
Upper basic/Secondary	30.5% (23.5% to –37.5%)	23.5% (16.9% to –30.1%)	31.8% (24.5% to –39.1%)	7.3% (3.6% to –11.0%)	6.9% (3.3% to –10.4%)
College/University	37.6% (25.3% to –49.9%)	16.1% (6.1% to –26.1%)	24.0% (12.6% to –35.4%)	11.3% (3.0% to –19.6%)	11.0% (2.1% to –19.9%)
Facility type					
Rural	25.7% (11.1% to –40.2%)	15.7% (7.1% to –24.3%)	23.4% (14.5% to –32.3%)	9.6% (2.4% to –16.8%)	25.7% (10.5% to –40.9%)
Urban	29.0% (23.7% to –34.2%)	26.7% (21.4% to –32.0%)	27.8% (22.4% to –33.2%)	10.7% (7.2% to –14.2%)	5.9% (2.8% to –8.9%)
Hospital	41.0% (28.4% to –53.5%)	8.8% (0.2% to –17.5%)	9.6% (2.4% to –16.8%)	26.7% (21.4% to –32.0%)	8.8% (0.2% to –17.5%)
Province					
Eastern	16.3% (6.9% to –25.7%)	26.2% (15.9% to –36.4%)	42.5% (30.4% to –54.7%)	11.9% (4.0% to –19.9%)	3.1% (0.0% to –7.8%)
Lusaka	36.1% (29.4% to –42.8%)	23.4% (17.7% to –29.0%)	22.3% (16.4% to –28.1%)	9.6% (5.8% to –13.4%)	8.6% (4.3% to –13.0%)
Southern	16.2% (7.4% to –25.0%)	27.5% (16.9% to –38.1%)	33.9% (23.1% to –44.7%)	14.7% (7.2% to –22.2%)	7.7% (1.8% to –13.6%)
Western	29.7% (17.7% to –41.8%)	27.4% (13.8% to –41.0%)	33.9% (20.1% to –47.8%)	5.4% (0.0% to –11.7%)	3.5% (0.0% to –8.6%)

LTFU, loss to follow-up.

### Association Between Latent Class and Care Status

**FIGURE 3.**
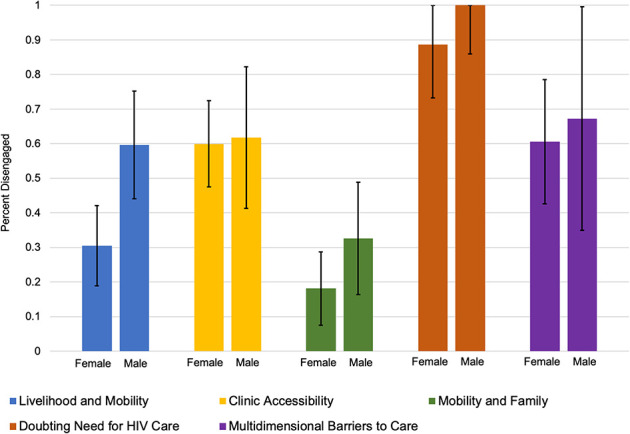
Estimated prevalence of disengagement by latent class and sex (n = 547). Estimated prevalence of disengagement based on marginal estimates from an adjusted Poisson regression that included an interaction term latent class and sex. Regression model incorporated population-representative sampling weights after tracing a random sample of patients who were considered lost to follow-up as of July 31, 2015. The *P*-value for the interaction term was 0.246.

Latent class membership was strongly associated with patients' current care status (ie, whether they remained out of care or had silently transferred to a new facility) in both unadjusted and adjusted analyses. The “Doubting Need for HIV Care” class had the highest prevalence of being out of care [97.9% (95% CI: 84.2% to –100.0%)] followed by those in the “Multidimensional Barriers to Care” class [62.8% out of care (95% CI: 44.2% to –81.3%)] and the “Clinic Accessibility” class [62.4% out of care (95% CI: 51.0% to –73.8%)]. In contrast, patients in the “Livelihood and Mobility” class [43.6% (95% CI: 34.5% to –52.7%) out of care] and “Mobility and Family” class [23.5% (95% CI: 14.4% to –32.6%) out of care] were more likely to have silently transferred a new facility (Table 5). Among the “Livelihood and Mobility” and “Mobility and Family” classes, the association with care status differed across sex, with males having a trend towards an increased proportion out of care, though this interaction was not statistically significant overall (*P* = 0.246) (Fig. 3).

**TABLE 5.** Association Between Latent Class Membership and Being Out of Care

Association Between Latent Class Membership and Being Out of Care, n = 547						
	Unadjusted		*P*	Adjusted		*P*
	Prevalence	Risk Difference		Prevalence	Risk Difference	
Latent class						
Livelihood and mobility	45.6% (35.3% to –55.8%)	REF	<0.001	43.6% (34.5% to –52.7%)	REF	<0.001
Clinic accessibility	62.0% (51.2% to –72.7%)	16.4% (1.5% to –31.2%)		62.4% (51.0% to –73.8%)	18.7% (3.8% to –33.7%)	
Mobility and family	22.6% (13.6% to –31.6%)	−23.0% (−36.6% to −9.4%)		23.5% (14.4% to –32.6%)	−20.1% (−33.0% to −7.2%)	
Doubting need for HIV care	94.0% (85.4% to –100%)	48.4% (35.0% to –61.7%)		97.9% (84.2% to –111.6%)	54.3% (37.0% to –71.6%)	
Multidimensional barriers to care	64.6% (45.1% to –84.1%)	19.0% (−3.0% to 41.0%)		62.8% (44.2% to –81.3%)	19.2% (−1.9% to 40.2%)	
Sex						
Female	44.5% (37.3% to –51.6%)	REF	0.030	43.0% (36.8% to –49.2%)	REF	0.005
Male	56.7% (48.2% to –65.3%)	12.3% (1.1% to –23.4%)		59.7% (50.3% to –69.0%)	16.7% (4.6% to –28.8%)	
Age at LTFU						
<25 yrs old	34.6% (20.1% to –49.1%)	REF	0.14	39.0% (24.0% to –54.0%)	REF	0.15
25–35 yrs old	55.2% (46.5% to –63.9%)	20.6% (3.7% to –37.5%)		56.8% (47.9% to –65.7%)	17.8% (1.1% to –34.5%)	
35–50 yrs old	49.9% (41.5% to –58.3%)	15.3% (−1.5% to 32.1%)		46.6% (38.9% to –54.2%)	7.5% (−9.8% to 24.9%)	
>50 yrs	38.0% (18.1% to –57.9%)	3.4% (−21.2% to 28.0%)		44.6% (26.0% to –63.1%)	5.5% (−18.6% to 29.7%)	
Enrollment CD4 count						
<200 cells/μL	49.9% (41.0% to –58.8%)	REF	0.90	44.8% (37.4% to –52.2%)	REF	0.24
200–350 cells/μL	54.5% (43.7% to –65.3%)	4.6% (−9.4% to 18.6%)		51.2% (41.3% to –61.1%)	6.4% (−6.2% to 19.0%)	
350–500 cells/μL	55.0% (39.7% to –70.3%)	5.1% (−12.6% to 22.9%)		59.5% (44.4% to –74.7%)	14.7% (−2.6% to 32.1%)	
>500 cells/μL	53.4% (36.1% to –70.6%)	3.5% (−16.0% to 22.9%)		57.2% (42.5% to –71.9%)	12.4% (−4.5% to 29.2%)	
WHO stage						
I	49.1% (40.8% to –57.4%)	REF	0.96	48.3% (40.6% to –55.9%)	REF	0.94
II	52.1% (38.4% to –65.7%)	3.0% (−13.0% to 18.9%)		48.4% (36.8% to –60.0%)	0.1% (−13.6% to 13.8%)	
III	52.3% (41.4% to –63.3%)	3.2% (−10.5% to 17.0%)		52.2% (41.7% to –62.8%)	3.9% (−10.3% to 18.2%)	
IV	52.3% (28.7% to –75.9%)	3.2% (−21.8% to 28.2%)		52.9% (29.6% to –76.2%)	4.6% (−20.9% to 30.1%)	
Time to LTFU						
<6 mo	48.2% (38.5% to –57.9%)	REF	0.55	44.2% (34.2% to –54.2%)	REF	0.48
6 months-2 yrs	53.5% (43.3% to –63.6%)	5.3% (−8.8% to 19.3%)		50.7% (41.6% to –59.8%)	6.5% (−6.8% to 19.8%)	
2–5 yrs	52.0% (40.7% to –63.3%)	3.8% (−11.1% to 18.7%)		57.3% (44.9% to –69.8%)	13.1% (−4.8% to 31.0%)	
>5 yrs	40.8% (26.9% to –54.6%)	−7.4% (−24.3% to 9.4%)		47.6% (32.0% to –63.2%)	3.3% (−16.5% to 23.2%)	
Prior episodes of LTFU						
0	49.9% (42.3% to –57.4%)	REF	0.99	52.1% (44.4% to –59.8%)	REF	0.89
1	49.8% (39.8% to –59.7%)	−0.1% (−12.6% to 12.4%)		47.4% (38.4% to –56.4%)	−4.7% (−17.4% to 7.9%)	
2	48.2% (31.7% to –64.6%)	−1.7% (−19.8% to 16.4%)		47.7% (31.3% to –64.0%)	−4.5% (−23.6% to 14.7%)	
>3	49.9% (25.9% to –73.9%)	0.0% (−25.1% to 25.1%)		46.7% (22.3% to –71.0%)	−5.5% (−31.5% to 20.5%)	
Marital status						
Single	51.6% (39.0% to –64.1%)	2.7% (−11.8% to 17.3%)	0.53	54.8% (43.7% to –65.9%)	5.6% (−7.8% to 19.0%)	0.29
Married	48.8% (41.5% to –56.2%)	REF		49.2% (42.3% to –56.0%)	REF	
Divorced	57.1% (43.0% to –71.1%)	8.2% (−7.6% to 24.1%)		54.7% (41.7% to –67.6%)	5.5% (−9.6% to 20.5%)	
Widowed	40.0% (22.4% to –57.6%)	−8.8% (−27.9% to 10.3%)		35.6% (20.7% to –50.5%)	−13.6% (−29.9% to 2.7%)	
Education						
None	62.1% (35.7% to –88.4%)	REF	0.063	74.0% (52.9% to –95.1%)	REF	0.007
Lower–Mid basic	59.1% (50.0% to –68.2%)	−3.0% (−30.9% to 24.9%)		56.5% (48.0% to –65.1%)	−17.5% (−40.0% to 5.1%)	
Upper basic/Secondary	45.2% (37.0% to –53.4%)	−16.9% (−44.5% to 10.7%)		47.9% (40.1% to –55.6%)	−26.1% (−49.2% to −3.1%)	
College/University	41.5% (27.4% to –55.6%)	−20.6% (−50.4% to 9.3%)		37.2% (25.1% to –49.3%)	−36.8% (−60.3% to −13.3%)	
Facility type						
Rural	44.2% (32.5% to –56.0%)	−9.8% (−23.4% to 3.8%)	0.021	43.4% (32.5% to –54.2%)	−7.8% (−20.0% to 4.3%)	0.41
Urban	54.1% (47.3% to –60.8%)	REF		51.2% (45.6% to –56.8%)	REF	
Hospital	36.1% (26.3% to –45.9%)	−18.0% (−29.9% to −6.0%)		44.8% (33.6% to –55.9%)	−6.5% (−19.1% to 6.1%)	
Province						
Eastern	43.0% (32.3% to –53.8%)	−12.6% (−25.6% to 0.3%)	0.007	46.0% (35.4% to –56.7%)	−7.5% (−20.6% to 5.6%)	0.039
Lusaka	55.7% (48.5% to –62.9%)	REF		53.5% (47.0% to –60.1%)	REF	
Southern	35.9% (25.5% to –46.3%)	−19.8% (−32.4% to −7.1%)		36.2% (27.3% to –45.0%)	−17.4% (−28.6% to −6.2%)	
Western	33.2% (18.4% to –48.0%)	−22.5% (−39.0% to −6.0%)		40.4% (24.4% to –56.3%)	−13.2% (−30.7% to 4.3%)	

LTFU, loss to follow-up.

## DISCUSSION

We used LCA methods to characterize 5 distinctive profiles of care disruptions based on the types and number of patient-reported reasons for LTFU in a population-representative sample of patients LTFU from HIV care in Zambia. In our model, 30.6% of patients reported predominately work/school obligations and mobility/travel (“Livelihood and Mobility” group), 28.9% reported issues associated with attending clinic (“Clinical Accessibility” group), 21.9% reported mobility/travel, family obligations, and transport issues (“Mobility and Family” group”), 10.2% reported doubts regarding their HIV status or need to attend clinic (“Doubting need for HIV Care” group), and 8.3% reported numerous (mean 5.6) barriers across multiple domains (“Multidimensional Barriers to Care” group) as the reasons for their care disruption. Although there were some notable trends, baseline characteristics were not strongly associated with belonging to a particular class. However, patients' care disruption profiles were the factor most strongly associated with whether they remained out of care or had silently transferred to a new facility after LTFU. These findings highlight how identifying unique patient profiles can deepen our understanding of heterogeneity in patients' behaviors and care outcomes and inform more targeted strategies for improving retention in care.

The profiles of care disruptions that we identified among patients LTFU from HIV care have important and durable implications for optimizing the public health response to HIV. To date, many interventions strategies have only focused on addressing a single type of barrier at a time (eg, travel vouchers for transport-related barriers, decreasing visit frequency to reduce burden of accessing care, peer-navigators for psychosocial support).^4^ These strategies are likely to be effective in only a subset of the patient population, and there is thus increasing recognition that the next phase of the global response to the HIV epidemic will require more targeted approaches.^4,6,15^ Focusing on patient profiles that speak more to the underlying drivers of patient behaviors will be crucial when developing and implementing the next-generation of intervention strategies. First, these profiles highlight that more holistic strategies that seek to target the multiple barriers an individual may face, rather than individual ones, may be more effective at improving retention.^16^ Second, they establish that several distinct intervention strategies will need to be implemented in order to address the care needs of the entire population.^6^ For example, patients with competing work obligations may require increased flexibility and decreased burden for receiving care (ie, extending visit intervals). For others, travel and mobility are unavoidable and facilitating transfers (which are often associated with prolonged gaps in care^17^) so they are seamless is needed. Another large group will likely benefit from improving the patient-centeredness and overall experience of attending HIV clinics,^18^ while only a smaller group might may require more intensive interventions that focus on providing psychosocial support and counseling.^4^ Lastly, they suggest the need for different interventions may vary across patient characteristics or the stage at which a patient is in their care (eg, newer versus more established patients).^6^ These are actionable insights that remain durable for public health planning and should be integrated with data on patient preferences^19,20^. Additional research should now seek to elucidate what the most effective intervention strategies will be for different patient profiles—either through standard or more adaptive methods—as well as how to effectively target these different strategies.

Our analysis also highlights how LCA represents an innovative data-driven and patient-centric method for synthesizing high-dimensional data on behavioral determinants. Patients often experience multiple barriers concurrently and it is often the interplay between them—not just one barrier—that lead to disengagement.^11,21–23^ In our analysis, for example, although 34.2% of patients reported mobility/travel-related barriers to care, their patient characteristics and outcomes varied depending on what other barriers were also present (and thus to which latent class they belonged). Those who also reported work/school obligations (ie, “Livelihood and Mobility”) were more likely to be male, on ART for longer, but also with more prior episodes of LTFU, and from urban areas; those who also reported transportation and family obligations (ie, “Mobility and Family”) were more likely to be female with no prior LTFU; whereas those who reported multiple additional barriers (ie, “Multidimensional Barriers to Care”) also trended toward being male with multiple episodes of prior LTFU, but from rural areas. Depending on the overall barrier profile, outcomes varied significantly across these groups (43.6%, 23.5%, and 62.4% being out of care, respectively), but this association also potentially differed across sex for the “Livelihood and Mobility” and “Mobility and Family” class but not the “Multidimensional barriers to care.” LCA synthesizes data at the person-level (ie, identifying patient–level profiles or phenotypes), which enables results to capture potentially complex interactions with other determinants of patient behavior (as opposed to methods that focus only on individual barriers). This allows it to extend prior studies that identified associations only with individual barriers to care (or categories of barriers) and outcomes such as retention and mortality.^11,24,25^ For this reason, LCA is also often considered a patient-centered^26^—as opposed to variable-centered—analytic method, and has been used in several other studies to assess the interplay between multidimensional constructs such as HIV acquisition risk,^27–31^ health care seeking behaviors,^32,33^ and engagement patterns^34,35^ at the person-level. Ultimately, the use of LCA should be extended as it can synthesize highly dimensional data to provide a more comprehensive and patient-centered understanding of the drivers of patient behaviors.

Leveraging patient profiles is also a promising strategy for differentiating between higher- and lower-risk patients. Traditional approaches to risk stratification have largely focused on an individual sociodemographic (eg, age or sex), socioeconomic (eg, food insecurity), or behavioral (eg, stigma) risk factor, but these frequently only differentiate between groups with relatively small absolute differences in risk.^23^ For example, in our study, there was a 16.7% difference in prevalence of being out of care between sexes and a 17.8% difference across age groups. There was, however, up to a 70% difference in prevalence of being out of care across different profiles of care disruptions. Because these profiles synthesize multidimensional behavioral characteristics, they also can likely explain a substantially higher proportion of the heterogeneity in risk across patients. This also makes them vital components to risk stratification and clinical prediction models.^23^ Future studies, however, still need to longitudinally assess the association between these profiles and patient outcomes, particularly considering that patient profiles may change over time (eg, in response to life disruptions that may also introduce new barriers to accessing HIV care^21,36–39^). Nevertheless, emphasizing more holistic patient profiles is an important advance to how we think about patient risk factors.

There are several limitations of our study. First, our data were collected cross sectionally and data were only based on self-report. Thus, a patient's care status at that time (ie, out of care or silently transferred to a new facility) could have influenced the reasons they reported for their initial care disruption, and, because of logistical reasons, we did not independently verify that patients who reported transferring to a new facility had in fact done so. Second, our data were only among patients who were considered LTFU from their original clinic and did not include any patients who remained in care. Prospective studies in a more generalized population are needed to confirm the association between patient profiles and outcomes. Third, our patient population also only included adults from the general HIV clinic population. Thus, our findings do not necessarily extend to other key populations of interest, such as adolescents and children, sex workers, intravenous drug users, or men who have sex with men. Additional studies, however, should leverage this approach for identifying profiles more relevant to these populations. Fourth, because of the large number of unique reasons patients reported, we conducted an initial round of data reduction based to combine similar reasons into a limited number of categories that could then be analyzed with LCA. This was done systematically based on theory, literature review, and contextual experience. Finally, it is important to note that the patient profiles that were identified are not necessarily immutable properties and only represent data-driven attempts to characterize patients' subgroups based on the available data. However, model diagnostics indicated a very good fit for the data with clear differentiation between latent classes, results were consistent in sensitivity analyses^12,14^ (Tables 4 and 5, Supplemental Digital Content, http://links.lww.com/QAI/B555), and our results do comport with the existing literature on barriers to engagement in HIV care.^11,25^

We used LCA and patient-reported reasons for care disruptions to identify and characterize 5 distinctive profiles of care disruptions that were strongly associated with patients' current care status in a population-representative sample of patients LTFU from HIV care in Zambia. These results underscore the importance of characterizing heterogeneity between patients in a holistic manner using data-driven methods such as LCA. Ultimately, these findings can be used to develop and implement more deliberate and targeted interventions strategies to improve retention in care for the diverse patient populations in public health HIV programs.
